# Bienzymatic Biosensor for Rapid Detection of Aspartame by Flow Injection Analysis

**DOI:** 10.3390/s140101028

**Published:** 2014-01-09

**Authors:** Maria-Cristina Radulescu, Bogdan Bucur, Madalina-Petruta Bucur, Gabriel Lucian Radu

**Affiliations:** Centre of Bioanalysis, National Institute of Research and Development for Biological Sciences, 296, Splaiul Independentei, Bucharest 060031, Romania; E-Mails: bucurica@yahoo.com (B.B.); madalina_dondoi@yahoo.com (M.-P.B.); rglucian2000@yahoo.com (G.L.R.)

**Keywords:** alcohol oxidase, carboxyl esterase, flow injection analysis, aspartame

## Abstract

A rapid, simple and stable biosensor for aspartame detection was developed. Alcohol oxidase (AOX), carboxyl esterase (CaE) and bovine serum albumin (BSA) were immobilised with glutaraldehyde (GA) onto screen-printed electrodes modified with cobalt-phthalocyanine (CoPC). The biosensor response was fast. The sample throughput using a flow injection analysis (FIA) system was 40 h^−1^ with an RSD of 2.7%. The detection limits for both batch and FIA measurements were 0.1 μM for methanol and 0.2 μM for aspartame, respectively. The enzymatic biosensor was successfully applied for aspartame determination in different sample matrices/commercial products (liquid and solid samples) without any pre-treatment step prior to measurement.

## 1. Introduction

In the last decades, the high-intensity sweeteners have increasingly been used by the food and pharmaceutical industries to improve the taste of different products. Despite the long-term usage of artificial high-intensity sweeteners like aspartame, saccharin, neotame, acesulfame potassium and sucralose, their safety is still debated and currently European Food Safety Agency (EFSA) is conducting a full re-evaluation process of aspartame under the mandate of the European Commission [1]. Toxicological and clinical studies indicate that an excess of artificial sweeteners induces various health problems such as memory loss, headaches, seizures, cancer, *etc.* [2–4]. In consequence, the analysis of sweeteners in foods and pharmaceutical preparations is important for health consumer protection.

Various analytical techniques have been applied in the analysis of natural sugars and artificial sweeteners. High performance liquid chromatography (HPLC) is widely used for the determination of sweeteners [5–7], but this technique is based on expensive equipment, requires long and complex sample pretreatment, uses toxic organic solvents and various reagents. Different alternative analytical methods based on various detections, such as electrochemical [8–10], spectrophotometric [11,12], chemiluminescent [13] or colorimetric detection [14,15] have been developed. Even if these techniques require simple equipment, some of them are time-consuming, involve different chemical reagents, or do not have the necessary selectivity for the analyte determination in relevant commercial samples.

(Bio)sensors are interesting analytical devices with good analytical performance for the rapid analysis of complex samples [16,17]. Only few papers describe biosensors for the determination of aspartame in soft drinks. Those biosensors were based on the chemical co-immobilization of enzymes on different electrodes, such as ammonia-gas-sensing electrode [18], platinum-based hydrogen peroxide electrode [19], oxygen electrode [20], or graphite epoxy composite electrode [21]. Another strategy is based on the enzyme immobilization into columns integrated in flow systems: two enzyme columns containing peptidase and aspartate aminotransferase, respectively, immobilized on activated aminopropyl glass beads and an L-glutamate oxidase electrode [22] or another system consisting of a column containing pronase and an L-amino acid oxidase electrode [23]. These biosensors require long analysis times, show a reduced linear range, short lifetimes, or weak detection limits. Thus, fast, inexpensive methods of analysis with improved selectivity and sensitivity are required to monitor sweeteners in an extensive range of different commercial product matrices.

This paper presents a rapid, simple, inexpensive, and stable bienzymatic amperometric biosensor based on co-immobilization of AOX and CaE on cobalt-phtalocyanine modified screen-printed electrodes for the fast and selective aspartame quantification in complex samples. The proposed biosensor was implemented in a FIA system that requires only minimum operator intervention. The FIA system was used for the determination of aspartame in commercial pharmaceutical formulations and food without any pretreatment other than sample solubilisation/dilution with a buffer solution.

## 2. Experimental Section

### 2.1. Chemicals and Materials

Alcohol oxidase AOX (from *Hansenula sp.* 7.7 UI/mg solid), carboxyl esterase CaE (from porcine liver, 17 UI/mg solid), aspartyl phenylalanine methyl ester (aspartame), bovine serum albumin BSA, *g*lutaraldehyde GA (25% solution in water), potassium phosphate monobasic, sodium phosphate dibasic and potassium chloride were purchased from Sigma-Aldrich (St.Louis, MO, USA). Methanol was obtained from Merck (Darmstadt, Germany). Standard solutions of methanol and aspartame were prepared daily in water.

The amperometric measurements were made with a 0.1 M phosphate buffer solution (PBS) pH 7.3 supplemented with 0.05 M potassium chloride. All aqueous solutions were prepared with purified water (18 MΩ·cm^−1^; Millipore, Billerica, MA, USA). The soft drinks samples were purchased from a local supermarket. The pharmaceutical formulations were obtained from a local pharmacy.

### 2.2. Instruments

All amperometric measurements were performed using a PGSTAT302N potentiostat/galvanostat (Metrohm-Autolab, Utrecht, The Netherlands) controlled by a PC with the software Nova 1.8. The electromagnetic noise produced by magnetic stirring during batch measurements was reduced using the filter from the ECD module set to 1 s. Cobalt-phthalocyanine (CoPC) screen-printed electrodes were kindly provided by BIOMEM-University of Perpignan, France [24]. An Ag/AgCl pseudoelectrode and a carbon auxiliary electrode were printed alongside with the working electrode. The monocanal FIA manifold was constructed with: a Gilson Minipuls 3 peristaltic pump (Gilson, Middleton, WI, USA), a methacrylate wall-jet flow cell (DropSens, Oviedo, Spain) for electrodes, a sample injection valve (Omnifit, Cambridge, UK) with a 50 μL sample loop, connectors and PTFE tubing with 1 mm i.d.

### 2.3. Biosensor Preparation

The methanol biosensors were prepared as follows: 7.7 IU of AOX was dissolved in 20 μL PBS and mixed with 5 μL of 0.6% BSA and 5 μL of 1.5% glutaraldehyde. Four μL of the resulting solution was carefully spread on the surface of the working electrode. The aspartame biosensors were prepared using a similar procedure based on a bienzymatic solution containing 7.7 IU AOX and 18.7 IU CaE in the 20 μL PBS. The electrodes were dried at room temperature for 1 h and then were stored in sealed plastic bags at −20 °C.

### 2.4. Amperometric Measurements

**Scheme 1.**
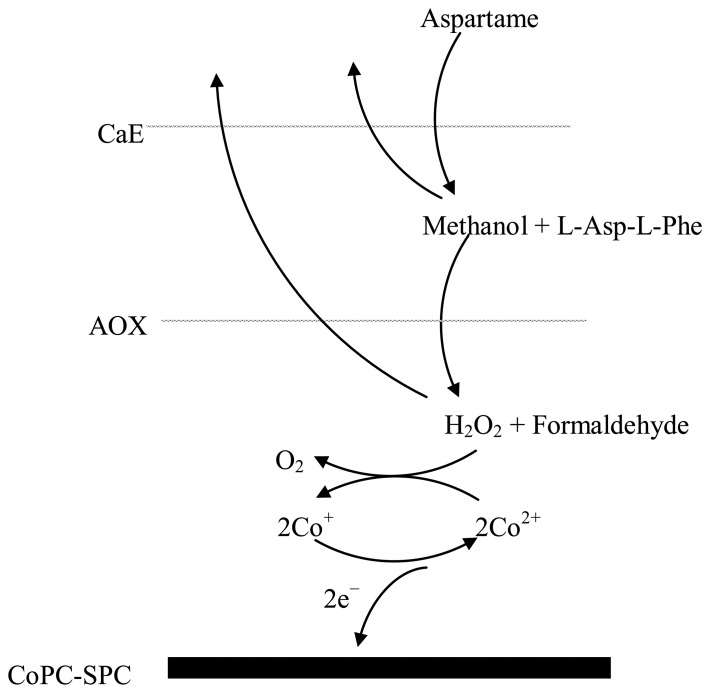
The aspartame detection mechanism. Note that a part of the enzymatically produced methanol and hydrogen peroxide diffuses towards solution and is not oxidized at the electrode surface.

Aspartame is first cleaved by carboxyl esterase in methanol and a dipeptide: L-Asp-L-Phe. The produced methanol is oxidized by AOX and the resulting hydrogen peroxide is quantified amperometrically (Scheme 1). The batch amperometric measurements were performed in a glass cell containing 5 mL of PBS under constant magnetic stirring. The current intensity was measured under a constant potential of +600 mV *vs.* the screen-printed Ag/AgCl pseudoreference electrode. Approximately 60 s are necessary for the baseline stabilization. After the sample injection in the cell the signal was recorded and a plateau was reached in 20 s, but the total analysis time includes also the cellule replacement and biosensor washing with distilled water. The analytical signal is the difference between the current intensities of the plateau and baseline. To reduce the errors and the analysis time the developed biosensor was used also in a FIA system. The sample was injected in the PBS carrier flow 0.2 mL/min using the valve and transported to the flow cell.

## 3. Results and Discussion

### 3.1. Biosensor Optimization

**Figure 1.**
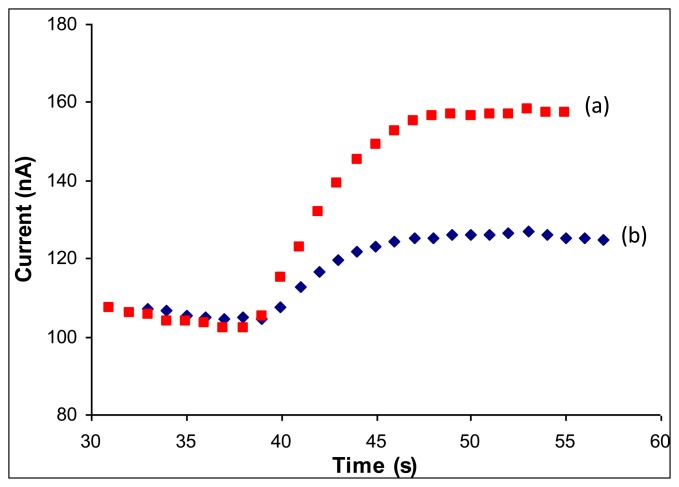
Analytical signals of the bienzymatic electrode recorded in batch for a concentration of 20 μM methanol (**a**) and 20 μM aspartame (**b**), respectively, under the optimum working conditions.

The initial optimization was carried out in batch mode. The biosensor was tested for both methanol and aspartame detection in order to optimize the working conditions and investigate the kinetics of the mono and bi-enzymatic processes. The optimum volume of enzymes/GA solution that covers the entire working electrode area is 4 μL. The optimum final concentrations of GA (0.25%) and BSA (0.1%) were chosen to obtain a stable immobilization of the enzymes. A decrease of the analytical signal was observed for higher concentrations of BSA and/or GA. The cobalt-phthalocyanine mediator can be used for hydrogen peroxide detection at different potentials ranging from +450 mV [25] to +600 mV [26]. The higher potentials allow better measurements sensibility, but are prone to interferences. We have chosen the working potential of 600 mV in order to achive a high measurement sensitivity, but for specific samples the potential can be reduced. A study of the pH influence on the analytical signal obtained using the bi-enzymatic electrode was studied in the range of 6–8 and the optimum value was 7.3. The optimum working conditions for both CaE and AOX are similar and the enzymes may be used together. The rapid responses of the developed biosensor were registered in less than 20 s under the optimum working conditions for both methanol and aspartame as it shown in Figure 1.

The AOX activity was first optimized for methanol detection and a 1 IU of AOX was chosen in order to obtain a satisfactory measurement sensitivity (1.887 nA/μM methanol) and detection limit (0.1 μM methanol). The AOX activity is significantly lower in comparison with 20 IU AOX used in other papers [20,21]. The optimum CaE activity per electrode was chosen as 2.5 IU based on the satisfactory sensitivity (0.825 nA/μM aspartame) and detection limit (0.2 μM aspartame). The lower sensitivity obtained for aspartame detection in comparison with methanol detection is explained by the fact that not all the enzymatically produced methanol is further oxidized by AOX because a part of the methanol is diffusing toward electrochemical cell (Scheme 1). Nevertheless, the magnitude of the analytical signals is sufficient for the aspartame detection. Bare and monoenzymatic electrodes (modified only with CaE or AOX) do not respond to aspartame.

### 3.2. Analytical Figures of Merit

**Figure 2.**
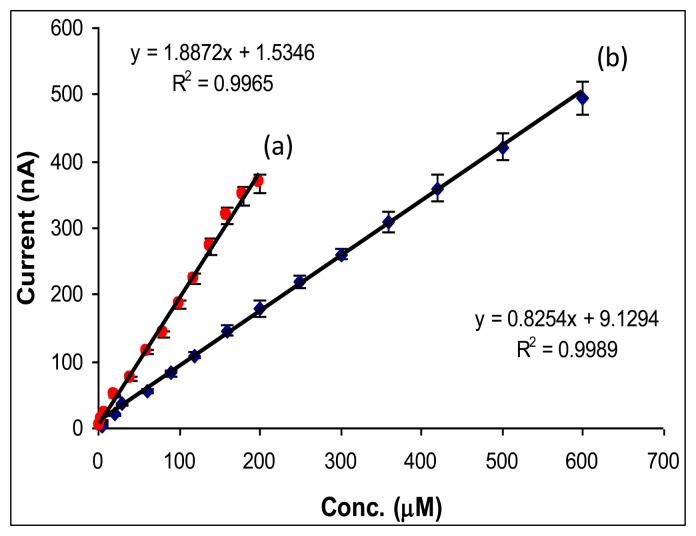
The calibration plots obtained for methanol (**a**) and aspartame (**b**) by using the bienzymatic biosensor in batch.

The biosensor response was studied in the concentrations range of 0–1,000 μM for methanol and aspartame, respectively. Under the optimum working conditions, a large linear domain was obtained for both aspartame and methanol (Figure 2). The calibration plot for aspartame determination in bach was characterized by a linear domain of concentrations between 5 and 600 μM with the equation I(nA) = 0.8254*x* Conc aspartame (μM) + 9.1294 and *R^2^* = 0.9989. The calibration plot for methanol determination was linear between 2 and 200 μM and was defined by the regression equation. I(nA) = 1.8872*x* Conc methanol (μM) + 1.5346 and R^2^ = 0.9965. The obtained detection limits (calculated as three times standard deviation of the blank) were 0.2 μM for aspartame and 0.1 μM for methanol, respectively.

The operational stability of a biosensor was measured by successive measurements of 20 μM aspartame and 20 μM methanol. The relative standard deviation (RSD) calculated for 10 successive batch measurements was 3.1% for methanol (53.4 nA ± 1.66) and 5.5% for aspartame (22.3 nA ± 1.45). More than 20 successive measurements can be performed with the same electrode without any significant decrease of the analytical signal. The reproducibility of the analytical signals recorded for the injection of 100 μM aspartame using different biosensors produced in the same lot was 6% (76.7 ± 4.58 nA, *n* = 6). The biosensors were stored in sealed plastic bags at −20 °C. A decrease of the signal up to 40% was observed after 1 month.

**Figure 3.**
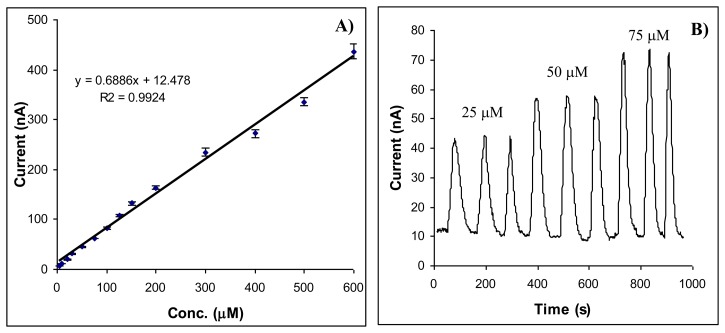
The calibration graph obtained for aspartame in FIA (**A**) and signals (**B**).

The calibration graphs for aspartame were similar in FIA and batch systems. The linear domain of concentrations was between 5 and 600 μM with the equation I(nA) = 0.6886x Conc aspartame (μM) + 12.478 and R^2^ = 0.9924 (Figure 3). The relative standard deviation for a concentration of 100 μM aspartame by using the FIA system was 2.7%, smaller than in batch. In the same time, the number of measurements was increased to 40 measurements/hour.

The performances of our biosensor are better or comparable with previous developed biosensors (Table 1). Our biosensor is simple, presents the shortest response time and has a good LOD and RSD. The obtained LOD is sufficient for the analysis of the expected aspartame concentrations in real food samples. The developed biosensor coupled with a simple FIA system has the advantages of a significant reduction of the analysis time in comparison with other systems (e.g., the biosensor developed by Odaci *et al.* requires 10 min [20], and of the reduction of the errors and the samples/reagents volume.

### 3.3. Interferences Study

**Figure 4.**
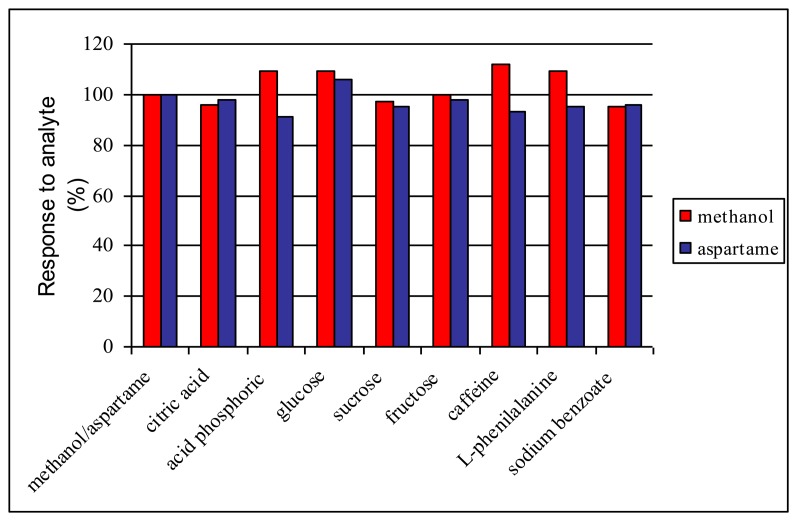
The relative response variation of the bienzymatic biosensor for a concentration of 20 μM methanol and 20 μM aspartame, respectively, in the absence and the presence of interferences.

Different potentially interfering substances e.g., citric acid, phosphoric acid, glucose, fructose, sucrose, caffeine, L-phenylalanine and sodium benzoate were investigated based on their possible presence in food and pharmaceutical samples. The initial study was carried out using an electrode without enzymes to measure the electrochemical interferences. Subsequently, the interferences study was carried out using bienzymatic biosensors for a methanol concentration of 20 μM, respectively aspartame and interference of 40 μM. When the interfering substance was added in the cell no analytical signal, was recorded. After the baseline stabilization, the methanol/aspartame concentration was added in the cell and the corresponding current value was registered. The biosensor response for methanol/aspartame was measured three times in the presence of each interfering substance and the mean value of the response variation was represented. The biosensor response varied between 94% and 112% in the presence of the studied interferences (Figure 4). These results must be compared to the measurement errors (5.5% mentioned in Section 3.2.) and demonstrate that the interferences have only minor effect on the biosensor's response.

### 3.4. Real Sample Analysis

The developed screen-printed biosensors were tested for the aspartame determination in different sample matrixes: soft drinks and commercial pharmaceutical formulations.

The soft drink brands, Coca-Cola and Pepsi Cola, were manufactured and marketed in Romania and can be classified in two categories: diet drinks and light drinks. These types of drinks mostly consist of carbonated water, sweeteners (aspartame and acesulfame-K), colorants, citric acid, sodium benzoate, phosphoric acid, food additives such as preservatives and flavors. The exact content of aspartame in such drinks was not mentioned on the label. Two pharmaceutical formulations of the same drug (Erdomed single dose sachet and bottle with granules for suspension) were analyzed. The samples contain the same active substance (erdosteine) and several excipients: aspartame, sugar, sodium benzoate, sodium amidonglycolate and flavors. The concentration of aspartame was labelled. The solid pharmaceutical samples were first dissolved in 100 mL water. Then all the samples (drinks and pharmaceuticals) were analyzed after adequate dilution in PBS without preliminary preparation such as concentration or extraction. Three aliquots of each sample were analyzed and interpolated in the calibration graph. The results for the aspartame amount founded in real samples are given in the Table 2. The obtained concentration levels of aspartame in the analyzed drink samples were found to vary between 58 and 152 mg/L and they did not exceed the maximum legally allowed UE concentration of 600 mg/L [1]. In addition, the concentrations of aspartame founded in commercial pharmaceutical formulations were in good agreement with those declared by the producer. The samples were spiked with 50 μM aspartame and the obtained recoveries were in the range of 94.1%–106.2%.

## 4. Conclusions

A rapid and reliable bienzymatic biosensor based on the enzymes AOX and CaE was developed for aspartame determination. The detection limits were 0.1 μM for methanol and 0.2 μM for aspartame, respectively. An improvement of the analysis time and measurement errors was achieved by coupling the biosensor with a simple FIA system. The response of the biosensor was very fast, 20 s are necessary to obtain the maximum response for methanol and aspartame concentrations, respectively. The developed system was successfully applied to the aspartame determination content in complex samples: soft drinks and commercial pharmaceutical formulations without the pre-treatment of the samples prior the analysis.

## Figures and Tables

**Table 1.** Comparison of various enzymatic biosensors for aspartame.

**Enzymes**	**Linear Range (μM)**	**LOD (μM)**	**RSD (%)**	**Analysis Time (min)**	**References**
Carboxyl esterase and alcohol oxidase	0.05–0.4	-	2.4	10	[20]
Carboxyl esterase and alcohol oxidase	2.5–400	-	3.9	3	[21]
Peptidase, aspartate aminotransferase and glutamate oxidase	Up to 1,000	20	2.2	7–8	[22]
Chymotrypsin and alcohol oxidase	85–1,200	7.3	3.0	4	[27]
Aspartame hydrolyzing enzyme, aspartate aminotransferase and glutamate oxidase	200–1,500	150	-	2-3	[28]
Peptidase, aspartate aminotransferase and glutamate oxidase	Up to 200	25	4.6	10–30	[29]
Carboxyl esterase and alcohol oxidase	5–600	0.2	5.5	Less than 2 (for FIA)	This work

**Table 2.** Analysis of aspartame in real samples.

**Sample**	**Aspartame Concentration (mg/L) ± SD**^a^	**Label Claimed Concentration (mg/L)**	**Recovery of Spiked Aspartame (%)**
Pepsi max classic	0	-	96.82
Pepsi max no sugar	129 ± 5.2	n.l b	103.77
Pepsi light	152 ± 7.0	n.l.	104.13
Coca-cola classic	0	n.l.	94.86
Coca-cola light	82 ± 3.1	n.l.	103.54
Coca-cola zero	58 ± 2.2	n.l.	106.21
Erdomed 175	7,512 ± 288	8,000	94.63
Erdomed 225	466 ± 19	500	96.13

Note:

a All results are expressed as average ±standard deviation of three determinations;

b n.l., not labeled.
